# Inferring pseudogene–MiRNA associations based on an ensemble learning framework with similarity kernel fusion

**DOI:** 10.1038/s41598-023-36054-y

**Published:** 2023-05-31

**Authors:** Chunyan Fan, Mingchao Ding

**Affiliations:** 1 School of Computer Science and Engineering, Xi’an Technological University, Xi’an, 710021 China; 2 School of Computer Science, Hubei University of Technology, Wuhan, 430068 China

**Keywords:** Computational biology and bioinformatics, Machine learning

## Abstract

Accumulating evidence shows that pseudogenes can function as microRNAs (miRNAs) sponges and regulate gene expression. Mining potential interactions between pseudogenes and miRNAs will facilitate the clinical diagnosis and treatment of complex diseases. However, identifying their interactions through biological experiments is time-consuming and labor intensive. In this study, an ensemble learning framework with similarity kernel fusion is proposed to predict pseudogene–miRNA associations, named ELPMA. First, four pseudogene similarity profiles and five miRNA similarity profiles are measured based on the biological and topology properties. Subsequently, similarity kernel fusion method is used to integrate the similarity profiles. Then, the feature representation for pseudogenes and miRNAs is obtained by combining the pseudogene–pseudogene similarities, miRNA–miRNA similarities. Lastly, individual learners are performed on each training subset, and the soft voting is used to yield final decision based on the prediction results of individual learners. The *k*-fold cross validation is implemented to evaluate the prediction performance of ELPMA method. Besides, case studies are conducted on three investigated pseudogenes to validate the predict performance of ELPMA method for predicting pseudogene–miRNA interactions. Therefore, all experiment results show that ELPMA model is a feasible and effective tool to predict interactions between pseudogenes and miRNAs.

## Introduction

Non-coding RNAs (ncRNAs) refer to the RNA molecules that could not translate into proteins, which composed up to about 98% of the human genome. These ncRNAs play an essential role in epigenetic regulation of gene expression at transcriptional and post-transcriptional levels. Pseudogenes are defined as incomplete copies of genes that code for proteins, but lack of coding function. However, pseudogenes could be transcribed into ncRNAs and be considered as regulators in organisms. MicroRNAs (miRNAs) are a class of small, single stranded, non-coding RNAs, which are involved gene expression at post-transcriptional level^1^. By binding to targeting mRNAs, miRNAs cause degradation and translation repression of mRNAs^2^. The fine-tuning of gene regulation by pseudogenes and miRNAs has attracted attentions in many biological processes.

Pseudogenes and miRNA are essential components of competing endogenous RNAs (ceRNAs) network. ceRNA hypothesis is proposed to describe the interactions among ceRNAs members and miRNAs^3^. The ceRNAs members include pseudogenes, long noncoding RNAs (lncRNAs), circular RNA (circRNAs), and protein-coding RNAs, etc. The ceRNAs could form a ceRNA network modulate mRNA expression and regulate protein levels. Recent experimental results show that abnormal expression and dysregulations of both pseudogenes and miRNAs are related to complex diseases. For example, pseudogene GBAP1 contributes to the development and progression of gastric cancer by sequestering the miR-212-3p from binding to GBA^4^. Therefore, pseudogenes and miRNAs can interact with each other, which jointly associated the occurrence of human diseases. However, it is very laborious and time-consuming to verify the associations between pseudogenes and miRNAs through biological experiments. So reasonable and effective computational methods is urgently need to mine the associations between pseudogenes and miRNAs.

Identifying pseudogene–miRNA associations contribute to discover more biological mechanisms in biological process and disease states. Compared with biological methods, the computational approaches are less time consumption. In the area of miRNA research, mining the potential miRNA-disease associations is a high hop topic^5–8^. For example, RWRMMDA model is proposed to predict the miRNA-disease associations by integrating multiple similarities, which also used improved extended random walk with restart algorithm based on miRNA similarity-based and disease similarity-based heterogeneous networks^9^. Zhou et al.^10^ proposed GBDT-LR method to prioritize miRNA candidates for diseases by combining gradient boosting decision tree with logistic regression. Besides, a large number of computational models are also developed to forecast other ncRNA associations and disease-biomolecule associations, for example, predicting the lncRNA–miRNA^11,12^, circRNA–miRNA^13,14^, lncRNA–disease^15,16^, circRNA–disease^17–19^, drug–disease^20^ interactions. Motived by these ncRNA interaction prediction, Zhou et al.^21^ incorporates feature fusion and graph auto-encoder to predict pseudogene–miRNA associations. In the model, various perspective attribute information for pseudogenes and miRNAs is obtained as their similarity features, and graph auto-encoder is used to obtain the low-dimensional representation of nodes. Then, the low-dimensional vector is fed into Extreme Gradient Boosting (XGBoost) to predict the pseudogene–miRNA associations. Compared with these ncRNA-miRNA and ncRNA-disease association prediction, only one computational model is developed to predict pseudogene–miRNA associations. Therefore, it still exists some limitations for further improvement. Especially, there is an urgent need to develop more accurate and efficient computational methods to infer associations between pseudogenes and miRNAs.

In this study, an ensemble learning framework with similarity kernel fusion (SKF) method is developed to mine the pseudogene–miRNA associations, named ELPMA. First, GIP kernel similarity, hamming profile similarity, cosine similarity for pseudogenes and miRNAs is calculated based on the known pseudogene–miRNA associations. Then, pseudogene expression similarity and miRNA function similarity are computed based on the pseudogene expression profiles and miRNA–target information, respectively. Besides, the pseudogene similarities and miRNA similarities are fused using SKF method. Then, the feature representation of pseudogene–miRNA interactions is constructed by combing the pseudogene–pseudogene similarity, miRNA–miRNA similarity, and experimentally validated pseudogene–miRNA associations. Next, resampling method is used to build multiple different balanced pseudogene–miRNA association training subsets, which could reduce the bias of small-scale samples. Finally, individual learners are performed on each subset to obtain the primitive outcomes, and the soft voting is used to yield final decision based on the prediction results of individual learners. To assess the effectiveness of ELPMA model, five-fold cross validation is implemented applied to assess the prediction performance of our proposed method. As a result, the mean area under the ROC curve (AUC) and mean area under the precision-recall curve (AUPR) of ELPMA method achieved 0.9896 and 0.9913, respectively. According to comparison with other four methods, assessment results shown that ELPMA method obtain comparable performance. In the case studies, the predicted miRNAs for the three investigated pseudogenes are also used to validate the prediction performance of ELPMA method. All the results shown that our proposed model could serve as a recommendable tool for predicting pseudogene–miRNA associations.

## Materials and methods

### Gold standard data set

The pseudogene–miRNA associations are obtained from starBase v2.0, in which very high stringency of pseudogene symbol is selected^22^. After screening and removing redundancy, 1570 experimentally supported pseudogene–miRNA associations is sorted out, covering 318 pseudogenes and 260 miRNAs. In this study, a pseudogene–miRNA adjacency matrix *PM*(*i*, *j*) is constructed based on the validated associations between pseudogenes and miRNAs. If there is an association between pseudogenes *p*(*i*) and miRNAs *m*(*j*), *PM*(*i*, *j*) is assigned as 1, otherwise 0.

### Expression similarity for pseudogenes

The expression level of pseudogenes in various cancers and normal tissues is obtained from dreamBase database^23^. In dreamBase database, expression information of pseudogenes is selected as the characteristic information of pseudogenes. When two pseudogenes have a higher correlation score tend to be more similarity expressed. The pseudogene expression profiles are measures as follows:

1 $$ SP\_EP(m_{i} ,m_{j} ) = \frac{{\sum\nolimits_{k = 1}^{N} {(x_{k} - \overline{x})(y_{k} - \overline{y})} }}{{\sqrt {\sum\nolimits_{k = 1}^{N} {(x_{k} - \overline{x})^{2} \sum\nolimits_{k = 1}^{N} {(y_{k} - \overline{y})^{2} } } } }} $$

where *N* is the number of properties of the expression profiles, *x*_*k*_ and *y*_*k*_ denote the expression values in different cancers and normal tissues.

### Function similarity for miRNAs

Given that miRNAs targeting more of the same genes tend to be involved in similar biological function. The interactions between miRNA and target gene information are obtained from miRTarBase^24^. The miRNA–target interactions are employed to measure the miRNA function similarity for each pair of miRNAs. If two sets of target genes (say *G*_*i*_ and *G*_*j*_) respectively have relationship with miRNA *M*_*i*_ and miRNA *M*_*j*_, the miRNA function similarity is calculated as follows:

2 $$ SM\_FS(m_{i} ,m_{j} ) = \frac{{card(G_{i} \cap G_{j} )}}{{\sqrt {card(G_{i} )} \cdot \sqrt {card(G_{j} )} }} $$

where *G*_*i*_ and *G*_*j*_ represent the sets of target gene that related with miRNAs.

### GIP kernel similarity for pseudogenes and miRNAs

The GIP kernel similarity is applied to calculate the similarity between pseudogenes and miRNAs based on the known pseudogene–miRNA association adjacency matrix^25^. The GIP kernel similarity for pseudogenes can be calculated as follows:

3 $$ \begin{gathered} SP\_GIP(p(i),p(j)) = exp( - \gamma_{p} \parallel p(i) - p(j)\parallel^{2} ) \\ \gamma_{p} = \frac{1}{{(\frac{1}{{n_{p} }}\sum\limits_{i = 1}^{{n_{p} }} {\parallel p(i)\parallel^{2} )} }} \\ \end{gathered} $$

where *p*(*i*) represents the pseudogene interaction profiles, which is a binary vector that encode the interaction between pseudogene *i* and all miRNAs, i.e., the *i*-th row of the gold standard pseudogenes-miRNA adjacency matrix *PM*. The parameter *γ*_*p*_ controls the kernel bandwidth. *n*_*p*_ is the number of pseudogenes.

Similar to pseudogenes, the GIP kernel similarity for miRNAs is defined as:

4 $$ \begin{gathered} SM\_GIP(m(i),m(j)) = exp( - \gamma_{m} \parallel m(i) - m(j)\parallel^{2} ) \\ \gamma_{m} = \frac{1}{{(\frac{1}{{n_{m} }}\sum\limits_{i = 1}^{{n_{m} }} {\parallel m(i)\parallel^{2} )} }} \\ \end{gathered} $$

where *m*(*i*) represents the miRNA interaction profiles, which is a binary vector that encode the interaction between miRNA *i* and each pseudogene, i.e., the *i*-th column of adjacency matrix *PM*. The parameter *γ*_*m*_ is also used to control the kernel bandwidth. *n*_*m*_ is the number of miRNAs.

### Hamming profile similarity for pseudogenes and miRNAs

Given the length for a pair of vectors are same, hamming profile is the number of elements of which corresponding values are different. The higher Hamming profile value represents the two vector has lower similarity. Hamming profile similarity for pseudogenes is calculated as follows:

5 $$ SP\_HP(p_{i} ,p_{j} ) = 1 - \frac{{\left| {IP(p_{i} )! = IP(p_{j} )} \right|}}{{\left| {IP(p_{i} )} \right|}} $$

where *IP*(*p*_*i*_) is the *i*-th row of the pseudogene–miRNA adjacency matrix *PM*.

Similarly, the hamming profile similarity for miRNA is defined as follows:

6 $$ SM\_HP(m_{i} ,m_{j} ) = 1 - \frac{{\left| {IP(m_{i} )! = IP(m_{j} )} \right|}}{{\left| {IP(m_{i} )} \right|}} $$

where *IP*(*m*_*i*_) is the *i*-th column of the pseudogene–miRNA adjacency matrix *PM*.

### Cosine similarity for pseudogenes and miRNAs

Cosine similarity algorithm has been widely used in the collaborative filtering recommendation algorithm. Here, based on known pseudogene–miRNA associations, the similarity of pseudogenes *p*_*i*_ and *p*_*j*_ is defined as follows:

7 $$ \begin{gathered} SP\_\cos (p_{i} ,p_{j} ) = \frac{{MP(p_{i} ) \cdot MP(p_{j} )}}{{\left\| {MP(p_{i} )} \right\|\left\| {MP(p_{j} )} \right\|}} \hfill \\ SP\_\cos = (SP\_\cos (p_{i} ,p_{j} ))^{r * r} \hfill \\ \end{gathered} $$

where *r* represents the number of pseudogenes. The binary vector *PM*(*p*_*i*_) indicates whether exist an association between pseudogene *p*_*i*_ and each miRNA (the row *i* of the *PM* matrix, if *p*_*i*_ is related to miRNA, otherwise 0). Meanwhile, *SP_cos*(*p*_*i*_, *p*_*j*_) represents the cosine similarity between pseudogene *p*_*i*_ and *p*_*j*_. *SP_cos* is the pseudogene cosine similarity matrix.

Similarly, the cosine similarity of miRNA *m*_*i*_ and miRNA *m*_*j*_ is computed as follows:

8 $$ \begin{gathered} SM\_\cos (m_{i} ,m_{j} ) = \frac{{MP(m_{i} ) \cdot MP(m_{j} )}}{{\left\| {MP(m_{i} )} \right\|\left\| {MP(m_{j} )} \right\|}} \hfill \\ SM\_\cos = (SM\_\cos (m_{i} ,m_{j} ))^{n * n} \hfill \\ \end{gathered} $$

where *MP*(*m*_*i*_) denotes whether there is an association between miRNA *m*_*i*_ and each pseudogene (the column of *MP* matrix, if *m*_*j*_ is related to pseudogene, otherwise 0). *SM_cos*(*m*_*i*_, *m*_*j*_) is the cosine similarity between miRNA *m*_*i*_ and miRNA *m*_*j*_. The *SM_cos* is the miRNA cosine similarity matrix. *n* is the number of miRNAs.

### Integrated similarity by similarity kernel fusion method

In this study, four kinds of pseudogene similarities and five miRNA similarities are calculated. The integrated pseudogene similarity is measured by combining pseudogene expression similarity, pseudogene GIP kernel similarity, pseudogene hamming profile similarity, pseudogene cosine similarity. The integrated miRNA similarity is calculated by combining miRNA function similarity, miRNA GIP kernel similarity, miRNA hamming profile similarity and cosine similarity. Here, similarity kernel fusion method is used to fuse the four pseudogene similarities and five miRNA similarities^26^. Let *S*_*p,r*_ (*r* = 1,2,…,4) represents the four pseudogene similarities and *S*_*m,n*_ (*n* = 1,2,…,5) represents the five miRNA similarities, respectively.

Firstly, each original kernel for pseudogenes is normalized by Eq. (9).

9 $$ NS_{p,r} (p_{i} ,p_{j} ) = \frac{{S_{p,r} (p_{i} ,p_{j} )}}{{\sum\nolimits_{{p_{k} \in P}} {S_{p,r} (p_{k} ,p_{j} )} }} $$

where when *NS*_*p,r*_ satisfies $\sum\nolimits_{{c_{k} \in C}} {NS_{c,m} (c_{k} ,c_{j} )} = 1$, *NS*_*p,r*_ is the normalized pseudogene similarity.

Then, a sparse kernel for each pseudogene similarity is computed by Eq. (10).

10 $$ F_{p,r} (p_{i} ,p_{j} ) = \left\{ {\begin{array}{*{20}c} {\frac{{S_{p,r} (p_{i} ,p_{j} )}}{{\sum\nolimits_{{p_{k} \in N_{i} }} {S_{p,r} (p_{i} ,p_{k} )} }}} & {p_{j} \in N_{i} } \\ 0 & {p_{j} \notin N_{i} } \\ \end{array} } \right. $$

where *F*_*c,m*_ is a sparse kernel and it satisfies $\sum\nolimits_{{c_{j} \in C}} {F_{c,m} (c_{k} ,c_{j} )} = 1$.* N*_*i*_ is a set of *p*_*i*_’s neighbors including *c*_*i*_ itself.

Therefore, four pseudogene similarities could be computed as Eq. (11).

11 $$ \begin{array}{*{20}c} {SP_{p,r}^{t + 1} = \alpha (F_{p,r} \times \frac{{\sum\nolimits_{k \ne 1} {SP_{p,k}^{t} } }}{2} \times F_{p,r}^{T} ) + (1 - \alpha )(\frac{{\sum\nolimits_{k \ne 1} {SP_{p,k}^{0} } }}{2})} & {\alpha \in (0,1)} \\ \end{array} $$

where $SP_{p,r}^{t + 1}$ is the status matrix of *r*-th pseudogene similarity kernel after *t* + 1 iterations.$SP_{p,k}^{0}$ denotes the initial status of *S*_*p,k*_.

After* t* + 1 steps, the overall kernel for pseudogenes is calculated as Eq. (12).

12 $$ S_{p} = \frac{1}{{4}}\sum\limits_{r = 1}^{{4}} {SC_{p,r}^{t + 1} } $$

Finally, a weight matrix *w*_*p*_ is used to remove the noise in the matrix* S*_*p*_.

13 $$ w_{p} {(}p_{i} {,}p_{j} {) = }\left\{ {\begin{array}{*{20}c} {1} & {{\text{if }}p_{i} \in N_{j} {\text{ and }}p_{j} \in N_{i} } \\ {0} & {{\text{if }}p_{i} \notin N_{j} {\text{ and }}p_{j} \notin N_{i} } \\ {{0}{\text{.5}}} & {{\text{otherwise}}} \\ \end{array} } \right. $$

The fused pseudogene similarity is computed as Eq. (14).

14 $$ S_{p}^{*} = w_{p} \circ S_{p} $$

Similarly, the integrated miRNA similarity as *S*_m_^*^ is computed, in which involved five miRNA similarities to be fused.

### Ensemble learning framework with resampling method

**Figure 1:**
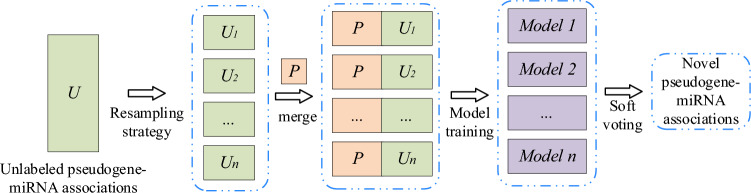
Ensemble learning framework for the pseudogene–miRNA association prediction.

To predict the potential pseudogene–miRNA associations, an ensemble learning framework with similarity kernel fusion method is proposed. Inspired by the previous research^27,28^, ELPMA model is proposed through the following steps: (1) using the resampling method to obtain multiple different training subsets, and the diversity of individual learners is increased; (2) to integrate the prediction results of individual learners, soft voting is employed to obtain the final prediction. The process of constructing the ensemble learning framework is shown in Fig. 1.

### Resampling strategy

There are 1570 experimentally confirmed pseudogene–miRNA associations as positive samples, and 81,110 unconfirmed pseudogene–miRNA pairs as unlabeled samples. So only a small part of experimentally confirmed pseudogene–miRNA associations. To settle the problem caused by the imbalanced dataset, the resample strategy is employed to build multiple different balanced training subsets. The negative samples are guaranteed to have the same number with positive samples. When construct a subset, all positive samples are sort out, and same unlabeled samples are randomly selected as negative samples. Then, the negative samples and positive training sample are combined to balance the positive and negative samples. The training set of positive sample *P* and the unlabeled sample set *U* are defined as follows:

15 $$ P = \{ p(i),m(j)||PM(p(i),m(j)) = 1\} $$

16 $$ U = \{ p(i),m(j)||PM(p(i),m(j)) = 0\} $$

where *P* represents the positive samples, and *U* denotes the unknown pseudogene–miRNA association samples.

In each training subset, the number of unlabeled pseudogene–miRNA associations is the same as the number of positive samples. The set *N *(*N* ∈ *U*) represents the negative samples selected from *U*, and the number of *N* is same as the number of *P*. The set of *T* = *P* ⋃ *N* is the training set in base learning.

### Sample representation

To learn the pseudogenes and miRNAs potential feature representation, multiple data source is incorporated to obtain the integrated similarities for pseudogenes and miRNAs. Here, a pseudogene–miRNA pair was taken as a sample. The feature vector of *i-*th pseudogene, *FP*(*p*(*i*)), is defined as follows:

17 $$ FP(p(i)) = (SP(p(i),p(1)),SP(p(i),p(2)), \ldots ,SP(p(i),p(N_{p} ))) $$

where *N*_*p*_ represents the number of pseudogenes. Similarly, the feature vector of *j*th miRNA, *FM*(*m*(*j*)), is defined as follows:

18 $$ FM(m(j)) = (SM(m(j),m(1)),SM(m(j),m(2)), \ldots ,SM(m(j),m(N_{m} ))) $$

where *N*_*m*_ represents the number of miRNAs. Then, the feature vector of each pseudogene–miRNA pair (*p*(*i*),*m*(*j*)) is defined by combining the *FP*(*p*(*i*)) and *FM*(*m*(*j*)) as follows:

19 $$ F(p(i),m(j)) = FP(p(i),FM(m(j)) $$

### Soft voting for pseudogene–miRNA association prediction

Ensemble learning combines multiple individual learners to increase the prediction performance compared to individual models. Owing to the training subsets are different and the feature spaces of the subsets are heterogenous, the trained individual learners are also different from each other. In this study, an ensemble learning framework is developed by using the XGBoost as individual learner on the multiple sample subsets. XGBoost is a machine learning algorithm in which regression trees is used as functions in gradient boosting to optimize trees^29^.

Set the output of a tree as shown below:

20 $$ f(x) = w_{q} (x_{i} ) $$

where *x*_*i*_ is the input vector, *q* represents the structure of each tree and *w*_*q*_ represents the score of the leaf node *q*. The output of the set of *K* trees is:

21 $$ \hat{y}_{i} = \sum\nolimits_{k = 1}^{K} {f_{k} (x_{i} )} $$

where *K* is the number of regression functions, the objective function for learning the set of *f*_*k*_ is shown as follows:

22 $$ \begin{gathered} L(\varphi ) = \sum\limits_{i = 1}^{n} {l(y_{i} ,\hat{y}_{i} )} + \sum\limits_{k = 1}^{K} {\Omega (f_{k} )} \hfill \\ \begin{array}{*{20}c} {where} & \Omega \\ \end{array} (f) = \gamma T + 0.5\lambda \left\| w \right\|^{2} \hfill \\ \end{gathered} $$

where *l* represents the loss function between the observed value *y*_*i*_ and predict value $\hat{y}_{i}$. Ω(*f*_*k*_) is the regularization term to avoid overfitting. *γ* is the pseudo-regularization hyperparameter. *λ* is the L2 norm for leaf weights.* T* is the total number of leaf nodes.

The optimal objective function value could be written as:

23 $$ \begin{gathered} \hat{L}^{(t)} (q) = - \frac{1}{2}\sum\limits_{j = 1}^{T} {\frac{{(\sum\nolimits_{i \in I} {g_{i} )^{2} } }}{{\sum\nolimits_{i \in I} {h_{i} + \lambda } }}} + \gamma T \hfill \\ \begin{array}{*{20}c} {} & {\begin{array}{*{20}c} {} & {} \\ \end{array} g_{i} = \delta_{{\hat{y}_{i}^{(t - 1)} }} l(y_{i} ,\hat{y}_{i}^{(t - 1)} )} \\ \end{array} \hfill \\ \begin{array}{*{20}c} {} & {} \\ \end{array} \begin{array}{*{20}c} {} & {h_{i} = \delta^{2}_{{\hat{y}_{i}^{(t - 1)} }} l(y_{i} ,\hat{y}_{i}^{(t - 1)} )} \\ \end{array} \hfill \\ \end{gathered} $$

where *I* is the set of leaf nodes, *g*_*i*_ is the first derivative of *l* and *h*_*i*_ is the second derivative of *l*.

Here, the outputs of XGBoost are taken as primitive results. Then, the soft voting is used to make the final decision. The prediction scores of individual learners are averaged, and confirmed whether the pseudogene is associated with each other. Take an unknown pseudogene–miRNA association as sample input, *n* individual learners could produce *n* prediction results, and then the *n* prediction results are integrated by using the soft voting strategy^30^. Specifically, the output of the *i-*th sample by soft voting is defined as follows:

24 $$ O(i) = \frac{1}{n}\sum\nolimits_{j = 1}^{n} {O(i,j)} $$

where *O*(*i*,*j*) is the prediction scores of the *j-*th individual learners for the *i-*th sample. *n* represents the number of training subsets. *O*(*i*) > 0.5 represents the pseudogene–miRNA pair is associated; otherwise, it is considered to be not associated with each other.

## Results

### Performance evaluation

In this work, *k*-fold cross validation is employed to evaluate the performance of the ELPMA model. The validated pseudogene–miRNA associations are regarded as the positive set, and equal number of samples are randomly selected from the negative sample set as negative samples. For each cross validation, (*k*-1) positive subsets and the same number of negative subsets took from *k* subsets to train the models; the remaining one positive subset and one negative subset are used for testing to evaluate the prediction performance. Specifically, fivefold and tenfold cross validation are used to evaluate the prediction performance of ELPMA model. Moreover, several metrics are used to measure the prediction performance of ELPMA method, including precision (Pre), sensitivity (Sen), accuracy (Acc), F1-score, AUC (Area under the receiver operating characteristic curve), AUPR (Area under the precision-recall curve), and MCC (Matthews’s correlation coefficient). The calculation formulas of these metrics are shown as follows:

25 $$ Pre = \frac{TP}{{TP + FP}} $$

26 $$ Sen = \frac{TP}{{TP + FN}} $$

27 $$ Acc = \frac{TP + TN}{{TP + TN + FP + FN}} $$

28 $$ F1 - score = \frac{2 \times Sen \times Pre}{{Sen + Pre}} $$

29 $$ MCC = \frac{TP*TN - FP*FN}{{\sqrt {(TP + FN)*(TP + FP)*(TN + FN)*(TN + FP)} }} $$

where *TP* and *TN* represent the number of true positives and true negatives, respectively. *FP* and *FN* represent the number of positives and negatives, respectively, that are wrongly predicted.

### Performance analysis of ELPMA method with different individual learners

To assess the ability of the ELPMA method to predict the associations between pseudogenes and miRNAs, fivefold cross validation is implemented on the gold standard data set. In the ensemble framework, different individual learners could affect the prediction performance. Here, AdaBoost, Random Forest (RF), Extreme Gradient Boosting (XGB) and Extremely Randomized Trees (ERT) are used as the individual learners, respectively. The individual learners are represented as ELPMA-AB, ELPMA-RF, ELPMA-XGB and ELPMA-ERT, respectively. In the ELPMA model, parameter selection are important factors, and the hyper-parameters of each model are tuned. For example, the number of individual learners of ELPMA is range from 2 to 20 with steps of 1. Furthermore, the range of hyper-parameter turning of ELPMA-XGB is as that n_estimators are selected from [50, 100, 200, 300, 400, 500], the learning rate is set from 0.1 to 0.9 with an interval of 0.1. The range of hyper-parameter turning of ELPMA-ERT is as that the value of max_depth is selected from [10, 20, 30, 40, 50] and the n_estimators are selected from [50, 100, 200, 300, 400, 500]. In addition, different hyper-parameters of ELPMA-AB and ELPMA-RF model are selected to obtain optimal performance. Finally, the prediction performance of the ELPMA model that using different individual learners is listed in Table 1. When the number of individual learners, n_estimators, learning rate are respectively set as 10, 400, 0.2, ELPMA-XGB yields the Precision of 0.9716, the Recall of 0.9369, the F1-score of 0.9540, the Acc of 0.9548, the AUC of 0.9897, the AUPR of 0.9914. As shown in Table 1, ELPMA-XGB is higher than other models in these seven metrics.

**Table 1:** The prediction performance of ELPMA model using different individual learners.

Model	Precision	Sensitivity	F1-score	Acc	AUC	AUPR	MCC
ELPMA-AB	0.7118	0.7153	0.7128	0.7124	0.7822	0.8000	0.4257
ELPMA-RF	0.9362	0.8592	0.8959	0.9003	0.9568	0.9664	0.8035
ELPMA-ERT	0.9650	0.8962	0.9292	0.9318	0.9793	0.9832	0.8660
ELPMA-XGB	**0.9716**	**0.9369**	**0.9540**	**0.9548**	**0.9897**	**0.9914**	**0.9102**

Significant values are in bold.

**Figure 2:**
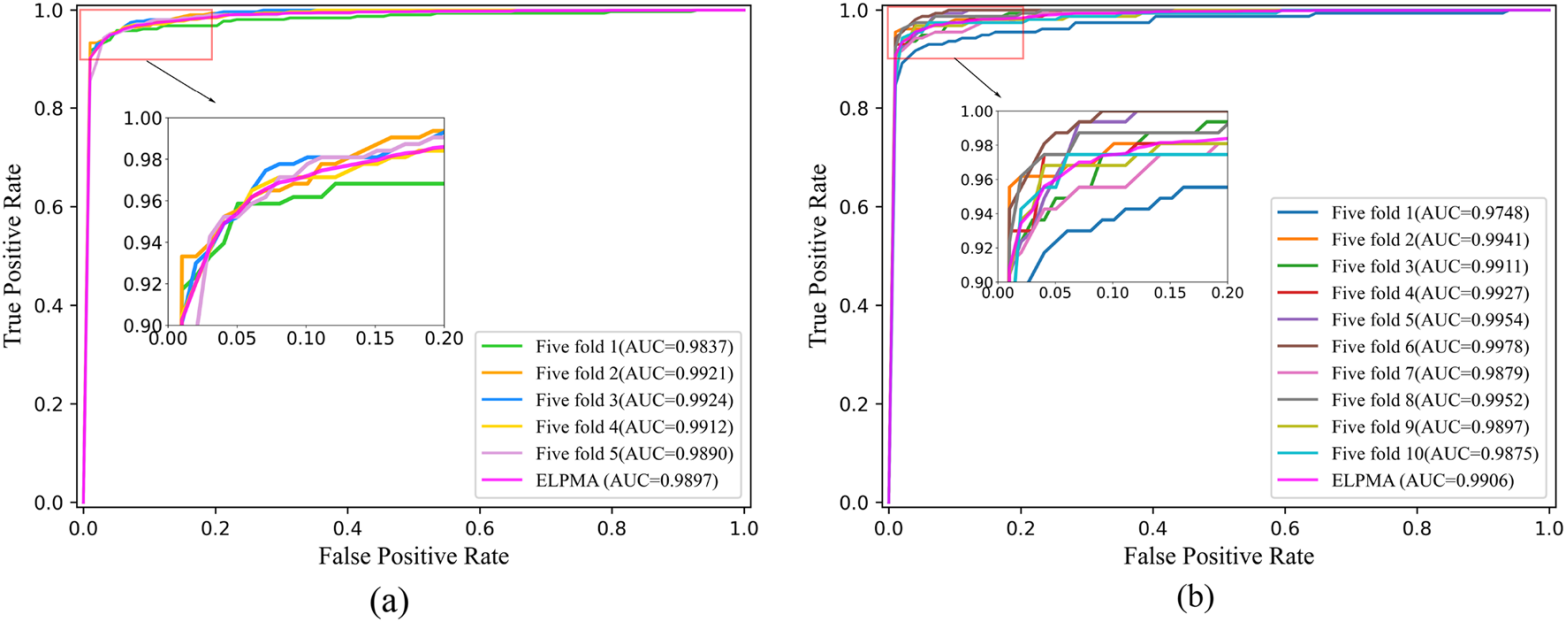
ROC curves under *k*-fold cross validation performed by the ELPMA-XGB framework. (**a**) ROC curves under fivefold cross validation; (**b**) ROC curves under tenfold cross validation.

In addition, the ROC curves of the *k*-fold cross validation are plotted by the proposed ELPMA-XGB method, respectively. The experimental results show that ELPMA-XGB achieves mean AUC values of 0.9897 and 0.9906 for the fivefold and tenfold cross validation (Fig. 2). Therefore, ELPMA-XGB model is appropriate as the individual learners of ELPMA method for the prediction of pseudogene–miRNA associations.

### Influence of training data on model performance

In the task, experimentally validated pseudogene-miRNA associations are selected as the only information source for model construction. The number of known pseudogene-miRNA associations may influence the prediction of our method ELPMA. To evaluate the impact of the number of training data on the performance, we used different proportions of training data to implement the ELPMA model. The fivefold and tenfold cross-validation results obtained by ELPMA is shown in Table S1. The results shown that the performance of ELPMA model getting better with the training data increasing. Therefore, the size of the training data has a great influence on the prediction performance of ELPMA model. With the number of training data increasing, the prediction performance of is also increased.

### Effectiveness of soft voting for the ensemble learning framework

**Figure 3:**
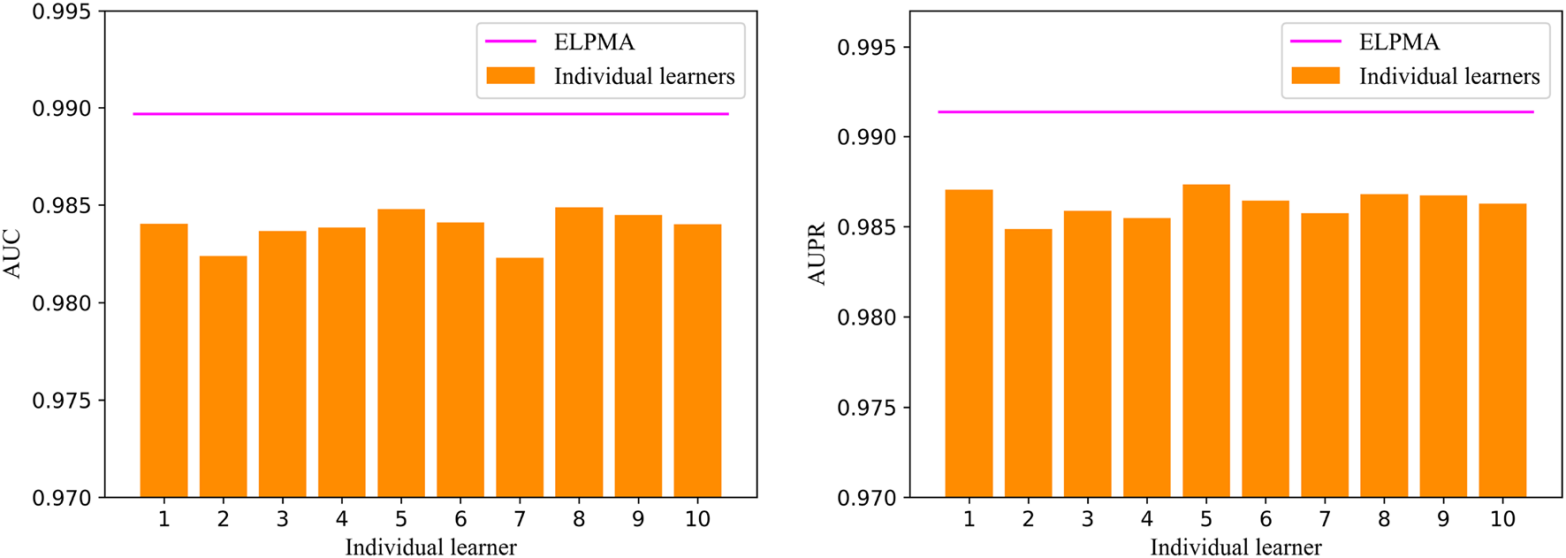
Performance comparison of ELPMA method and individual learners.

To demonstrate the effectiveness of the soft voting for the ensemble learning method, the soft voting performance is compared with individual learners on ELPMA model. Detailed results of the comparison are shown in Fig. 3. In the figures, the horizontal axis represents the index number of individual learners, and the vertical axis are the AUC values and AUPR values. From the Fig. 3, we also seen that the AUC of individual learners is between 0.9823 and 0.9849, and the AUPR of individual learners is between 0.9849 and 0.9873 under fivefold cross validation. The results indicate that soft voting in the proposed method could improve the prediction performance of ELPMA model. It also indicates that ELPMA is an effective framework to predict the pseudogene–miRNA interactions.

### Comparison with other existing methods

**Figure 4:**
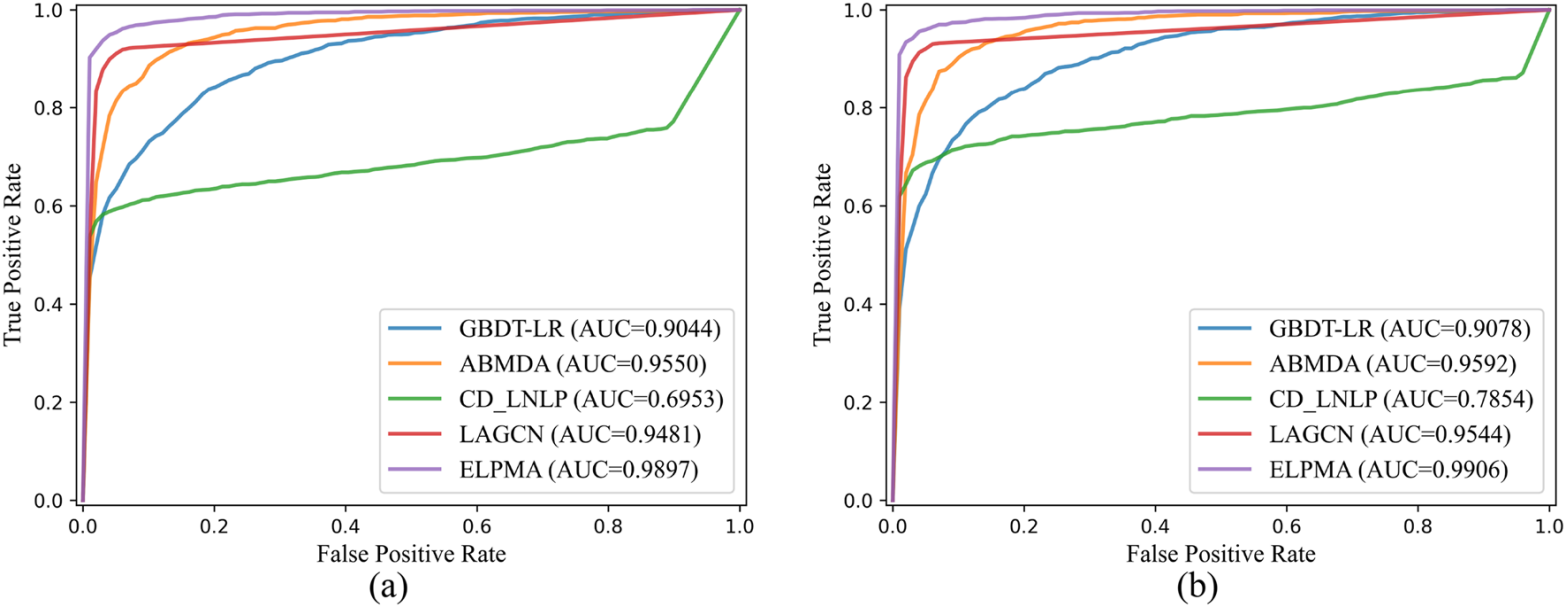
ROC curves of different methods under *k*-fold cross validation. (**a**) ROC curves under fivefold cross validation; (**b**) ROC curves under tenfold cross validation.

To comparatively illustrate the superiority of ELPMA method, GBDT-LR^10^, ABMDA^31^, CD_LNLP^17^, and LAGCN^20^ are compared with ELPMA method to predict the pseudogene–miRNA interactions. These five methods are individual evaluated based on gold standard data set with *k*-fold cross validation and recommended hyperparameters. As show in Fig. 4, ELPMA shows the best performance in term of the average AUC values under fivefold and tenfold cross validation. It shows that the ROC curves of ELPMA model is above those of GBDT-LR, ABMDA, CD_LNLP and LAGCN method in most cases. The average AUC scores of ELPMA method are up to 0.9897 and 0.9906 for the fivefold and tenfold cross validation, respectively, which is superior to the other four methods (Fig. 4). In addition, the results of performance evaluation indicators such as F1-score, Acc, MCC are shown in Table 2 for fivefold and tenfold cross validation. Although the Precision of ELPMA is inferior to ABMDA and Acc of ELPMA is inferior to CD_LNLP and LAGCN, the evaluation metrics of ELPMA are higher than others (Table 2). Furthermore, we used the paired *t*-test based on 10 runs of fivefold and tenfold cross-validation to test the performance of the ELPMA method and the comparison methods. Table 3 shows that ELPMA is significantly preferred to other computational methods in terms of Sensitivity, F1-score, AUC, AUPR and MCC (Table 3). Therefore, all the above results show that ELPMA method provides a great improvement in predict the pseudogene–miRNA interactions.

**Table 2:** Comparison with multiple evaluation metrics under fivefold and tenfold cross-validation.

	Model	Precision	Sensitivity	F1-score	Acc	AUC	AUPR	MCC
Fivefold cross-validation	GBDT-LR	0.8200	0.8166	0.8179	0.8176	0.9044	0.9144	0.6358
	ABMDA	**0.9832**	0.2834	0.4381	0.6411	0.9550	0.9519	0.3966
	CD_LNLP	0.7780	0.4822	0.5954	**0.9876**	0.6953	0.5216	0.6069
	LAGCN	0.1632	0.8076	0.2712	0.9832	0.9481	0.4847	0.3582
	ELPMA	0.9716	**0.9369**	**0.9540**	0.9548	**0.9897**	**0.9914**	**0.9102**
Tenfold cross-validation	GBDT-LR	0.8278	0.8306	0.8287	0.8275	0.9078	0.9145	0.6558
	ABMDA	**0.9848**	0.3478	0.5078	0.6728	0.9592	0.9551	0.4487
	CD_LNLP	0.8594	0.5605	0.6785	**0.9899**	0.7854	0.6264	0.6895
	LAGCN	0.1007	0.8261	0.1794	0.9853	0.9544	0.4633	0.2852
	ELPMA	0.9727	**0.9414**	**0.9565**	0.9573	**0.9906**	**0.9922**	**0.9155**

Significant values are in bold.

**Table 3:** The statistical results by paired *t*-test for ELPMA and other comparison methods.

ELPMA versus		GBDT-LR	ABMDA	CD_LNLP	LAGCN
Fivefold cross-validation	*p*-value of Precision	3.1222e−19	3.7527e−04	1.0708e−13	1.8361e−34
	*p*-value of Sensitivity	2.4777e−19	9.1047e−22	6.9457e−27	4.0794e−19
	*p*-value of F1-score	4.7760e−21	1.8721e−18	1.9262e−26	6.8365e−32
	*p*-value of Acc	8.4523e−21	2.0776e−21	1.6029e−19	2.0969e−18
	*p*-value of AUC	8.7304e−19	3.7505e−18	1.3119e−29	2.3623e−17
	*p*-value of AUPR	5.4014e−19	6.0815e−18	3.2105e−30	3.8908e−34
	*p*-value of MCC	9.5274e−21	6.4989e−21	3.2757e−23	1.8933e−30
Tenfold cross-validation	*p*-value of Precision	2.7171e−19	0.0018	3.1185e−10	3.7167e−38
	*p*-value of Sensitivity	4.5501e−20	2.907e−27	1.0613e−26	1.1716e−20
	*p*-value of F1-score	1.3765e−23	1.7704e−23	2.7238e−26	2.1321e−36
	*p*-value of Acc	8.2969e−23	1.9452e−26	3.0679e−19	1.0305e−17
	*p*-value of AUC	1.8574e−20	2.5035e−16	1.1239e−30	2.7548e−23
	*p*-value of AUPR	7.2994e−18	8.1945e−14	1.8536e−33	4.9185e−38
	*p*-value of MCC	8.1499e−23	2.0395e−25	3.2985e−22	2.4717e−33

### Case studies

To illustration the prediction performance of ELPMA method in screening pseudogene–miRNA interactions, case studies of three pseudogene related miRNA are conduct for further validation. Given the investigated pseudogene–miRNA interaction to be unknown in all known associations. In this section, the pseudogene *MSTO2P*, *MTND4P12* related miRNAs are removed in the known associations, and then use other associations to train the model and predict the probability of all miRNAs associated with the investigated pseudogenes. Through the calculation of ELPMA method, the candidate associations between pseudogene and miRNAs are sorted in descending order. Then, the top 10 rank results are selected with high probability scores for the three investigated pseudogenes, and the predicted associations are verified with the starBase database.

Pseudogene *MSTO2P* is found to be implicated in several diseases including lung cancer^32^, colorectal cancer^33^, etc. *MSTO2P* could function as a miR-128-3p sponge in non-small cell lung cancer cells (NSCLC), and *MSTO2P*/miR-128-3p to regulate coptisine sensitivity of NSCLC cells via TGF-*β* pathway. In addition, *MSTO2P* related top 10 miRNAs, in which 9 of the top10 is proved by starBase (Table 4).

**Table 4:** The top 10 associated miRNAs for pseudogene MSTO2P, MTND4P12.

Pseudogene	Rank	miRNA	Evidence
MSTO2P	1	hsa-miR-20a-5p	starBase
	2	hsa-miR-106b-5p	starBase
	3	hsa-miR-93-5p	starBase
	4	hsa-miR-519d-3p	starBase
	5	hsa-miR-20b-5p	starBase
	6	hsa-miR-17-5p	starBase
	7	hsa-miR-106a-5p	starBase
	8	hsa-miR-128-3p	starBase
	9	hsa-miR-448	starBase
	10	hsa-miR-373-3p	Unconfirmed
MTND4P12	1	hsa-let-7b-5p	starBase
	2	hsa-miR-98-5p	starBase
	3	hsa-let-7e-5p	starBase
	4	hsa-let-7d-5p	starBase
	5	hsa-let-7a-5p	starBase
	6	hsa-let-7c-5p	starBase
	7	hsa-miR-4500	starBase
	8	hsa-miR-4458	starBase
	9	hsa-let-7g-5p	starBase
	10	hsa-let-7f-5p	starBase

*MTND4P12* is considered as an oncogenic pseudogene upregulated in skin cutaneous melanoma, and it can upregulate the expression of oncogene *AURKB* by serving as ceRNA^34^. Hsa-let-7e-5p is also identified as candidate miRNA that regulated by *MTND4P12,* hsa-let-7e-5p and *MTND4P12* is co-expression in skin cutaneous melanoma. As shown in Table 4, the *MTND4P12* related top 10 miRNAs is supported by starBase.

## Conclusion

Increasing evidences show that both pseudogenes and miRNAs play oncogenic or tumor-suppressive roles in disease progression. Predicting pseudogene–miRNA associations will contribute to understanding the pathological mechanisms, diagnosis, and treatment of diseases. In this work, a computational method is proposed to infer the associations between pseudogenes and miRNAs, which employed an ensemble learning framework with similarity kernel fusion, named ELPMA. By comparing with other four models, the prediction performance of our proposed method is powerful to predict the pseudogene–miRNA interactions. The case study of investigated *MSTO2P* and *MTND4P12* related miRNAs also proved the ELPMA method is reliable and effective.

The good performance of ELPMA method is attributed to three main factors: (1) ELPMA integrates the biological information including pseudogene expression profiles and miRNA–targets interactions. (2) ELPMA introduces the resampling method to settle the problem caused by the imbalanced pseudogene–miRNA dataset. (3) The application of XGBoost as individual learner of the ensemble learning framework guarantees the effectiveness of learning the meaning of combinations of features from feature representation.

There are also some limitations in the ELPMA method. First, the gold standard pseudogene-miRNA associations may have nosy, and the negative samples are randomly selected from the unconfirmed associations, limiting the prediction performance. In addition, the ELPMA method relies on the known pseudogene–miRNA interaction network, and it could not predict novel pseudogene-miRNA interactions without any known associations. Therefore, developing more effective framework is essential to infer the associations between pseudogenes and miRNAs.

## Supplementary Information

Supplementary Table S1.

## Data Availability

The data will be made available on request from the corresponding author.
