# Supplementary material for: Inferring pseudogene–MiRNA associations based on an ensemble learning framework with similarity kernel fusion
Source: Sci Rep. 2023 May 31;13:8833. doi: 10.1038/s41598-023-36054-y (PMC10232424; doi:10.1038/s41598-023-36054-y)
Supplement: Supplementary file 1 — Supplementary Table S1. [file 41598_2023_36054_MOESM1_ESM.docx]

Table S1. Results of 5-fold and 10-fold cross-validation achieved by ELPMA with different proportions of training data

|  | **Proportion (%)** | **Precision** | **Sensitivity** | **F1-score** | **Acc** | **AUC** | **AUPR** | **MCC** |
| --- | --- | --- | --- | --- | --- | --- | --- | --- |
| 5-fold cross-validation | 10 | 0.5381 | 0.4718 | 0.4960 | 0.5285 | 0.5239 | 0.6008 | 0.0593 |
|  | 20 | 0.6601 | 0.6594 | 0.6575 | 0.6594 | 0.7434 | 0.7923 | 0.3205 |
|  | 30 | 0.8189 | 0.7247 | 0.7685 | 0.7818 | 0.8433 | 0.8775 | 0.5678 |
|  | 40 | 0.8609 | 0.7771 | 0.8168 | 0.8257 | 0.8849 | 0.9088 | 0.6546 |
|  | 50 | 0.8964 | 0.8318 | 0.8627 | 0.8675 | 0.9196 | 0.9381 | 0.7372 |
|  | 60 | 0.9317 | 0.8365 | 0.8814 | 0.8875 | 0.9498 | 0.9592 | 0.7792 |
|  | 70 | 0.9356 | 0.8790 | 0.9061 | 0.9090 | 0.9629 | 0.9695 | 0.8199 |
|  | 80 | 0.9511 | 0.9060 | 0.9278 | 0.9295 | 0.9731 | 0.9749 | 0.8604 |
|  | 90 | 0.9643 | 0.9151 | 0.9390 | 0.9406 | 0.9800 | 0.9836 | 0.8823 |
|  | 100 | **0.9716** | **0.9369** | **0.9540** | **0.9548** | **0.9897** | **0.9914** | **0.9102** |
| 10-fold cross-validation | 10 | 0.5341 | 0.4908 | 0.5053 | 0.5287 | 0.5322 | 0.6286 | 0.0592 |
|  | 20 | 0.6747 | 0.6682 | 0.6698 | 0.6712 | 0.7407 | 0.7933 | 0.3441 |
|  | 30 | 0.8508 | 0.7414 | 0.7912 | 0.8050 | 0.8600 | 0.8892 | 0.6164 |
|  | 40 | 0.8716 | 0.7931 | 0.8297 | 0.8377 | 0.9051 | 0.9241 | 0.6791 |
|  | 50 | 0.9072 | 0.8510 | 0.8775 | 0.8816 | 0.9308 | 0.9470 | 0.7655 |
|  | 60 | 0.9328 | 0.8526 | 0.8903 | 0.8955 | 0.9647 | 0.9645 | 0.7947 |
|  | 70 | 0.9428 | 0.8962 | 0.9186 | 0.9208 | 0.9704 | 0.9727 | 0.8432 |
|  | 80 | 0.9558 | 0.9092 | 0.9318 | 0.9335 | 0.9786 | 0.9801 | 0.8683 |
|  | 90 | 0.9699 | 0.9250 | 0.9467 | 0.9480 | 0.9847 | 0.9873 | 0.8972 |
|  | 100 | **0.9727** | **0.9414** | **0.9565** | **0.9573** | **0.9906** | **0.9922** | **0.9155** |
